# NKX6.1 transcription factor: a crucial regulator of pancreatic β cell development, identity, and proliferation

**DOI:** 10.1186/s13287-020-01977-0

**Published:** 2020-10-29

**Authors:** Idil I. Aigha, Essam M. Abdelalim

**Affiliations:** 1 College of Health and Life Sciences, Hamad Bin Khalifa University (HBKU), Qatar Foundation (QF), Doha, Qatar; 2 Diabetes Research Center (DRC), Qatar Biomedical Research Institute (QBRI), Hamad Bin Khalifa University (HBKU), Qatar Foundation (QF), PO Box 34110, Doha, Qatar

**Keywords:** Diabetes, Transcription factor, β cell mass, Pluripotent stem cells, Pancreatic progenitors, Cell therapy

## Abstract

Understanding the biology underlying the mechanisms and pathways regulating pancreatic β cell development is necessary to understand the pathology of diabetes mellitus (DM), which is characterized by the progressive reduction in insulin-producing β cell mass. Pluripotent stem cells (PSCs) can potentially offer an unlimited supply of functional β cells for cellular therapy and disease modeling of DM. Homeobox protein NKX6.1 is a transcription factor (TF) that plays a critical role in pancreatic β cell function and proliferation. In human pancreatic islet, NKX6.1 expression is exclusive to β cells and is undetectable in other islet cells. Several reports showed that activation of NKX6.1 in PSC-derived pancreatic progenitors (MPCs), expressing PDX1 (PDX1^+^/NKX6.1^+^), warrants their future commitment to monohormonal β cells. However, further differentiation of MPCs lacking NKX6.1 expression (PDX1^+^/NKX6.1^−^) results in an undesirable generation of non-functional polyhormonal β cells. The importance of NKX6.1 as a crucial regulator in MPC specification into functional β cells directs attentions to further investigating its mechanism and enhancing NKX6.1 expression as a means to increase β cell function and mass. Here, we shed light on the role of NKX6.1 during pancreatic β cell development and in directing the MPCs to functional monohormonal lineage. Furthermore, we address the transcriptional mechanisms and targets of NKX6.1 as well as its association with diabetes.

## Introduction

Diabetes mellitus (DM), affecting millions of people worldwide, is a metabolic disease caused by the loss or impaired function of insulin-producing pancreatic β cells [1]. β cell replacement is a promising strategy for the treatment of diabetes. Transplantation of the whole pancreas or isolated islet cells obtained from cadavers has been suggested as an alternative therapeutic approach for diabetes. However, despite successfully reducing hyperglycemia in transplant recipient diabetic patients, there are major limitations in this approach such as the inadequate supply of human pancreata and the necessity for lifelong use of immunosuppressant drugs [2]. Understanding the molecular mechanisms underlying human pancreas development can potentially provide better strategies for treating DM.

Pancreatic islets consist of five types of endocrine cells including insulin (INS)-producing β cells, glucagon (GCG)-producing α cells, somatostatin (SST)-producing δ cells, pancreatic polypeptide (PP)-producing cells, and the ghrelin-producing ε cells [3]. β cells represent the largest number of cells within pancreatic islets. β cells constitute 80% of mouse islet cells and 50–60% of the human islet cells [4–6]. Human pancreatic islets have a lower percentage of β cells and a higher percentage of α and δ cells in comparison to mouse islets [4]. Within the pancreatic islets, there is an interaction between different endocrine cells, which may contribute in the diabetes pathogenesis. For example, it has been reported that the GCG secretion from α cells is inhibited by β cell hormone, INS. Also, GCG activates INS secretion by elevating cAMP levels in ß cells GCG. In addition, human α cells secrete acetylcholine, which enhances glucose-stimulated insulin secretion (GSIS) in ß cells [7–9]. δ cells secrete SST that has an inhibitory effect on α and ß cells [10]. α and ß cells secrete urocortin 3 (UCN3), which enhances the secretion of SST from δ cells [11]. Taken together, these findings indicate that the interaction between different islet cells is involved in the pathogenesis of diabetes development [12].

Multiple signaling pathways, growth factors, and transcription factors (TFs) are engaged in regulating pancreatic development through orchestrating the balance in differentiation, proliferation, and maturation of endocrine and exocrine cells comparting the pancreas [13]. One of the key TFs involved in early and late pancreatic β cell specification is the homeobox protein Nkx6.1 (NKX6.1) [13], which is also expressed in the nervous system during development and plays a key role in motor neuron specification [14].

Human pluripotent stem cells (hPSCs) including human embryonic stem cells (hESCs) and human induced PSCs (hiPSCs) have the ability to differentiate into all types of cells, which make them a renewable source for functional insulin-producing cells [15]. Furthermore, they are a promising reference for in vitro disease modeling and drug discovery [16]. Stepwise differentiation protocols simulating the human fetal pancreas development have been employed for differentiating hPSCs into insulin-producing cells in vitro [17, 18]. These studies indeed successfully reported the generation of functional monohormonal β cells in vitro, i.e., secreting only insulin and can respond adequately to glucose challenge. However, the existing differentiation protocols still require further improvements [13]. This is mainly due to the small numbers of generated insulin-producing cells and their poor efficiency in responding to high levels of glucose (reviewed in [13, 15]). Pancreatic and duodenal homeobox 1 (PDX1) and NKX6.1 are two main transcription factors (TFs) that are highly expressed in both multipotent pancreatic progenitor cells (MPCs) and functional β cells. hPSC-derived MPCs (hPSC-MPCs), co-expressing PDX1 and NKX6.1 (PDX1^+^/NKX6.1^+^), mature into functional β cells when transplanted into immune-deficient mice and successfully reduced their high glucose blood levels [15]. On the other hand, MPCs lacking NKX6.1 expression (PDX1^+^/NKX6.1^−^) develop into polyhormonal cells and fail to function properly in vivo, indicating that the expression of NKX6.1 has a crucial role in guiding MPC development into β cells [15]. Moreover, specification of MPCs into non-β endocrine lineage may require the suppression of NKX6.1.

Collectively, NKX6.1 has an indispensable role in the specification of MPCs into mature functional β cells. Furthermore, it plays a major part in maintaining the function of adult pancreatic β cells. In this review, we will thoroughly discuss NKX6.1 expression during different stages of pancreatic development in humans and mice, its interaction with other TFs involved in pancreatic β cell development, and its role in the pathogenicity of DM.

## Expression of NKX6.1 during pancreas development

During human development, early endodermal tissue becomes specified towards a pancreatic fate before evagination of dorsal and ventral pancreatic buds. At 4 weeks of gestation (G4w), the dorsal bud appears, followed by the ventral bud [19]. These buds are populated with MPCs. Signaling and transcriptional events then promote the MPCs towards acinar, endocrine, or ductal fates. Once specified, endocrine progenitors undergo further differentiation with the lineage-specific TFs promoting final differentiation and maturation steps [3]. All adult pancreatic cells are originated from the same MPCs that express a group of TFs, including PDX1, SOX9, FOXA2, NKX6.1, HNF6, and PTF1A [20, 21]. The orchestrated expression of key TFs is mandatory for the development of functional mammalian pancreas. PDX1, NKX6.1, NKX2.2, PAX6, NGN3, ISL1, and NEUROD1 are among the main TFs controlling the chronological development of the distinct types of endocrine cells comprising the islet of Langerhans [13]. The PDX1 and NKX6.1, two TFs expressed in the MPC stage, determine the generation of functional pancreatic β cells [22]. In humans, PDX1 expression begins at 4 weeks and later becomes restricted to pancreatic β cells [19, 23]. In the adult pancreas, PDX1 expression is not restricted to islet cells, where it is detected in the exocrine pancreas of humans [24, 25].

**Fig. 1:**
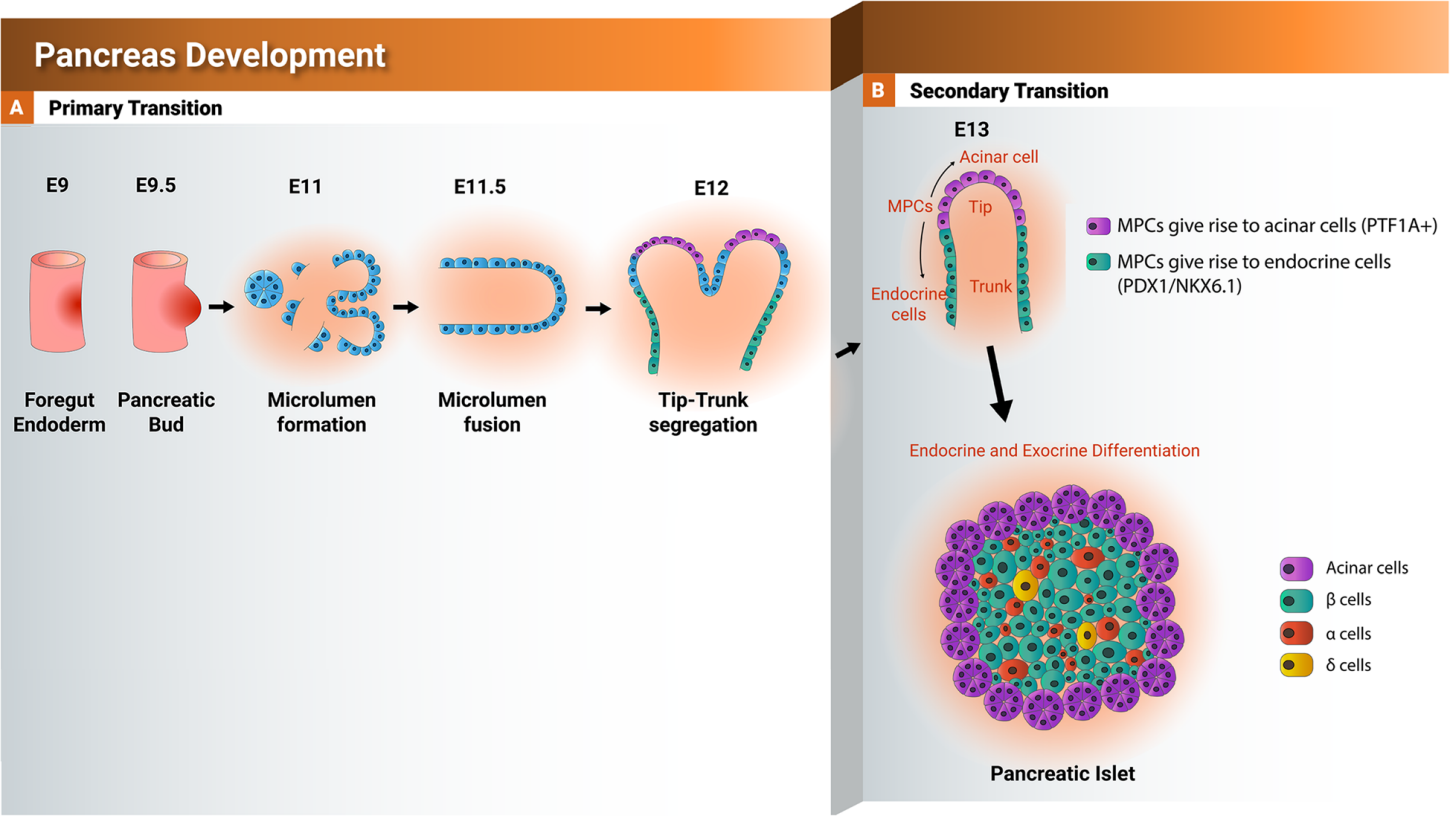
Schematic representation showing the development of the pancreas from foregut endoderm into pancreatic islet. Tip and trunk domain formation and segregation during the primary transition. During the secondary transition, the formed trunk domain (green) gives rise to the endocrine progenitors and subsequently pancreatic islets, while the tip domain (purple) develops to the exocrine progenitors expressing PTF1A

**Fig. 2:**
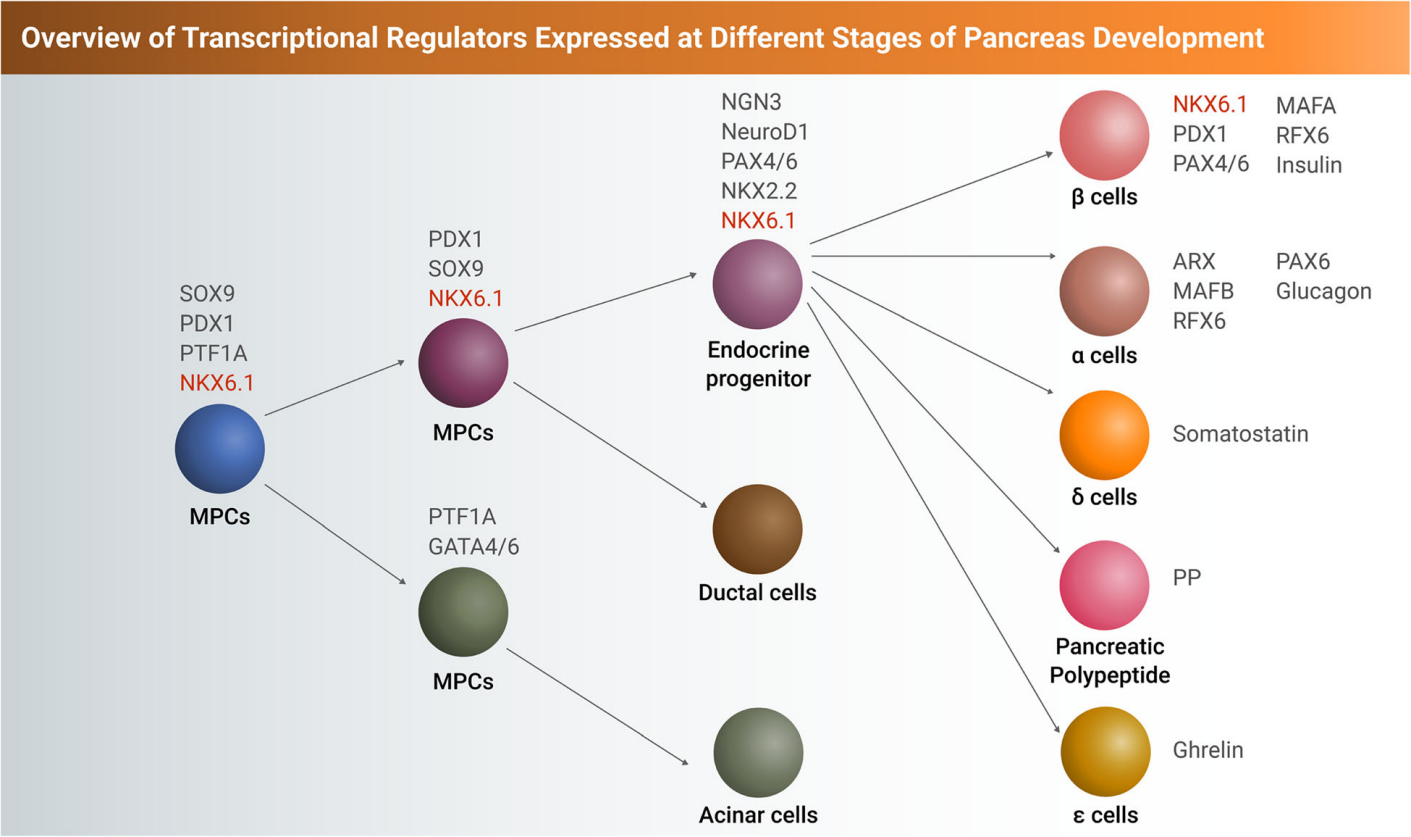
The expression of key transcription factors (TFs) during different stages of multipotent pancreatic progenitor (MPC) differentiation into different lineages of pancreatic cells. NKX6.1 expression starts at the MPC stage, continues in the endocrine lineage, and becomes restricted to β cells

NKX6.1, a homeobox-containing TF, was first identified and isolated from chromosome 4 of islet and insulinoma cell lines [26]. NKX6.1 is expressed at early stages of pancreatic development as well as in adult β cells, where it is involved in several functions during pancreatic development (Figs. 1 and 2). During pancreatic development, there are three different NKX6.1+ cell populations: pancreatic progenitor cells (PDX1^+^/NKX6.1^+^), endocrine progenitor cells (NGN3^+^/NKX6.1^+^), and pancreatic beta cells (insulin^+^/PDX1^+^/NKX6.1^+^) [27]. Throughout pancreas development, NKX6.1 expression increases in the trunk area of the pancreatic epithelium, which later specifies into endocrine lineage [13]. Nkx6.1 is initially expressed at mouse embryonic day 9.5 (E9.5) in the pancreatic epithelium and continues up to E13, where its expression becomes restricted to β cells. At E10.5 in mice, Nkx6.1 and Ptf1a are co-expressed in a large percentage of MPCs. By E12.5, Nkx6.1 and Ptf1a make their separate way as Nkx6.1 becomes exclusively confined in the trunk domain of the developing pancreas giving rise to the endocrine lineage, while Ptf1a becomes fully committed to exocrine fate originating from the tip domain [28]. Furthermore, few early GCG-expressing cells that do not co-express either PDX1 or NKX6.1 can be detected in the dorsal buds of the developing pancreas [29]. Some of these early Gcg^+^ cells have a low early insulin (Ins) expression by E11.5 and those Gcg^+^/Ins^+^ cells do not express any Nkx6.1 [29]. E13.5 marks the emergence of the first Ins-expressing cells that are negative for Gcg (Gcg^−^/Ins^+^). These cells display a strong expression of both Pdx1 and Nkx6.1 [30]. The Ins^+^/Gcg^+^ cells and the later appearing single Ins^+^ cells comprise two distinct populations of hormone expressing cells. It has been suggested that the Gcg/Ins co-expressing cells serve as a transition state for the mature β cells [31]. However, this theory has been rejected and instead it has been suggested that the mature β cells arise from non-hormone expressing pancreatic progenitor cells co-expressing PDX1 and NKX6.1 [32]. Around days E16.5 and E17.5, the first somatostatin (Sst)-expressing cells are observed. Although some of these cells co-express Pdx1, no Sst/Nkx6.1 co-expressing cells have been found at this stage. At E18.5, almost all Sst^+^ cells have Pdx1^+^ nuclei, while Sst^+^/Nkx6.1^+^ cells have not been detected. It is worth noting that after E18.5, the percentage of Pdx1^+^ nuclei in Sst-expressing cells remarkably decrease to 10–20%. By E20.5, pancreatic polypeptide (PP)-positive cells are detected [30]. Throughout the development of the PP-expressing cells, few cells are positive for Pdx1, but the expression of Nkx6.1 is extremely rare. The above observations provide strong evidence that Nkx6.1 is mainly expressed in β cell precursors and mature β cells in adult pancreas of rodents [30].

Pancreatic endocrine specifications are not identical in humans and rodents. The data available on the early development of human pancreas remain limited due to the difficulty in obtaining the human embryo at early stages. It has been reported that in mice, the polyhormonal cells appear before the development of monohormonal pancreatic β cells. In contrast, in humans, the monohormonal β cells appear prior to the appearance of polyhormonal cells during pancreas development [13, 19], suggesting the difference in the pancreatic β cell development between humans and mice. A previous study revealed that the phases of pancreas development between mouse and human are the same; however, there are differences in the timing of lineage commitments and expression of certain markers [19]. In humans, the NKX6.1 expression starts at 6.5 weeks of gestation (G6.5w) and increases exponentially at G13w only in insulin-producing cells [19]. In the mouse model, Nkx6.1 expression is absent in the MPCs giving rise to the acinar cells [28]. On the other hand, in humans, the MPCs giving rise to acinar cells express NKX6.1 along with acinar cell marker GATA4 in earlier stages (~G7w) before losing NKX6.1 expression from the peripheral cells of MPCs a week later [19]. A recent study examined the expression of NKX6.1, PTF1A, glycoprotein 2 (GP2), and INS in human neonatal pancreatic tissue at G33w, G37w, and G39.5w [33]. The immunostaining results showed the presence of two NKX6.1 populations: at the leading edge, the NKX6.1 is co-expressed with GP2 but negative for INS (NKX6.1^+^/GP2^+^/INS^−^), while in the center of the developing pancreas, there are islet clusters co-expressing NKX6.1 and INS without GP2 expression (NKX6.1^+^/INS^+^/GP2^−^) [33]. Furthermore, at G37w, NKX6.1 has been found to be co-expressed with PTF1A and GP2 (NKX6.1^+^/PTF1A^+^/GP2^+^) in the tip of the developing human pancreas; however, in the trunk of the developing human pancreas, only the expression of NKX6.1 is detected (NKX6.1^+^/PTF1A^−^/GP2^−^), which is similar to that seen in the mouse pancreas [33]. In the human pancreas, the NKX6.1 expression does not co-localize with SST, PP, or amylase during early or adult pancreas development reinforcing the notion of NKX6.1 constraint into insulin-producing β cells [19].

### Role of NKX6.1 during early pancreas development

**Fig. 3:**
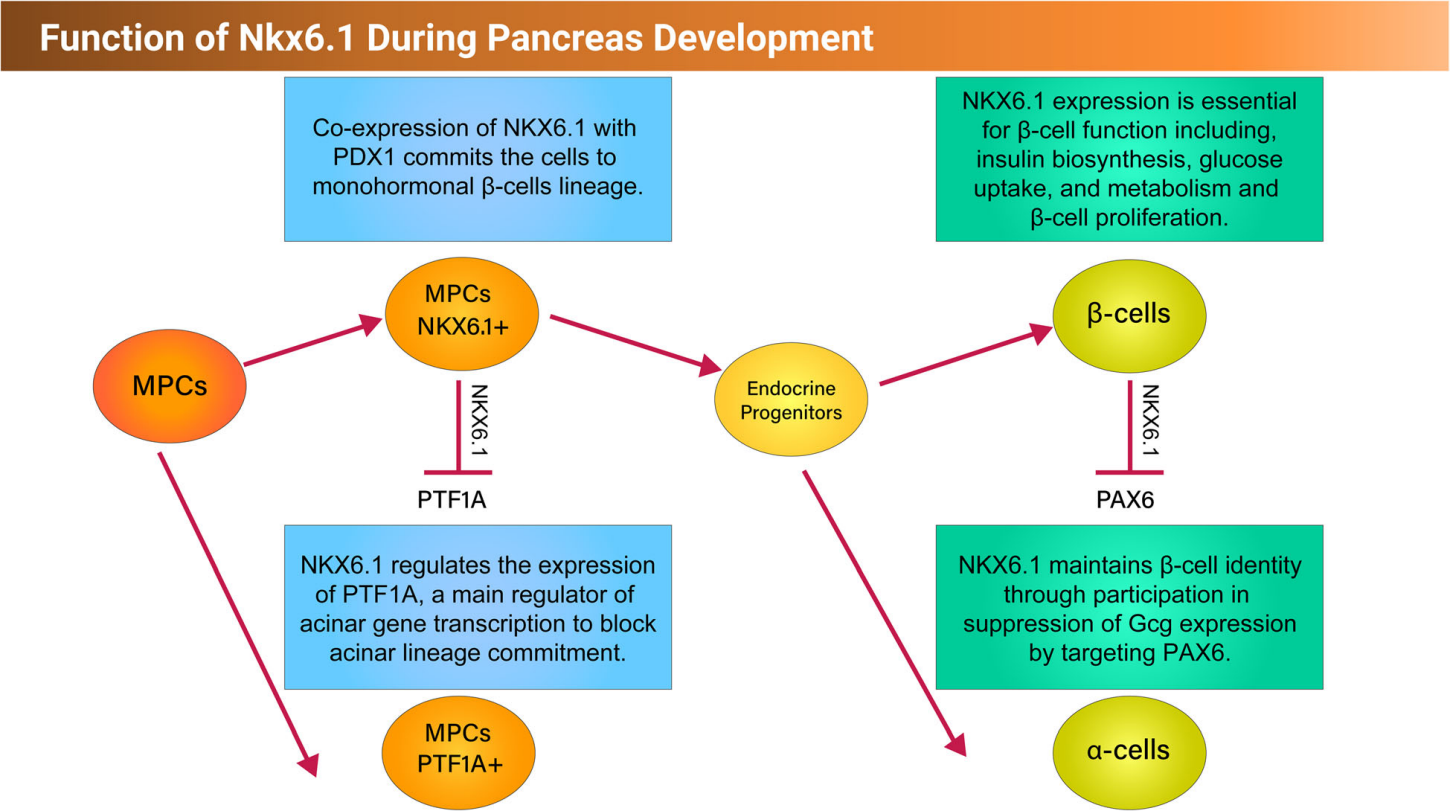
Schematic representation showing the function of NKX6.1 during early and late pancreatic development

NKX6.1 plays important roles during early and late stages of pancreatic development as well as in mature β cells. As described above, NKX6.1 expression starts during the MPC stage after PDX1 induction and its expression in PDX1+ cells determines pancreatic islet cell fate [34] (Fig. 3). In rodents and humans, glucose-responsive monohromonal β cells originate from MPCs co-expressing PDX1 and NKX6.1 (PDX1^+^/NKX6.1^+^) [13, 15]. In mice, deletion of *Nkx6.1* prevents the generation of functional monohormonal β cells; however, it allows the development of polyhormonal β cells [35]. Also, it has been shown that forced expression of Nkx6.1 in PDX1^+^ MPCs rescues pancreatic β cell development in *NKX6.1* mutant progenitors [36], highlighting the critical role of Nkx6.1 expression at the MPC stage in the development of β cells. However, Nkx6.1 ectopic expression in Ngn3^+^ cells at the endocrine progenitor (EP) stage is not sufficient to drive EPs to β cell fate, suggesting that Nkx6.1 expression is required before the induction of the endocrine program through Ngn3 expression [36]. While previous studies showcased that Ngn3 emerges from MPCs that express Nkx6.1 (Pdx1^+^/Nkx6.1^+^) or lack Nkx6.1 expression (Pdx1^+^/Nkx6.1^−^), other studies demonstrated that Ngn3 expression can precede that of Nkx6.1 during early pancreatic development [37]. It has been reported that the early induction of hormone cells intercepts with the development of functional β cells [32]. Altogether, the NKX6.1 activation in MPCs prior to NGN3 induction and the co-expression of NGN3 and NKX6.1 at the EP stage are crucial for their commitment to the desired functional β cell lineage [38].

Deciding between exocrine and endocrine pancreatic cell fates occurs at the early pancreatic progenitor stage and controlled by the balance between NKX6.1 and PTF1A (Fig. 3). Previous studies showed that there is an antagonist mechanism between NKX6.1 and PTF1A controlling cell fate determination. PTF1A, a crucial TF for exocrine pancreas development, is detected in early pancreatic progenitors giving rise to endocrine and exocrine fates [39]. *Nkx6.1*-deficient embryos showed a significant high expression of Ptf1a and a substantial reduction in Ngn3+ EPs [40]. On the other hand, overexpression of Nkx6.1 in the MPCs significantly reduces Ptf1a expression hence blocking acinar cell generation. Also, overexpression of Nkx6.1 in acinar cell line leads to a significant reduction in the Ptf1a expression. This effect is blocked when Nkx6.1 binding site in Ptf1a is mutated [40]. These results provide undeniable proof that Nkx6.1 directs the choice between endocrine and acinar lineages and that PTF1A, a main regulator of acinar gene transcription, is directly regulated by NKX6.1. Noticeably, the majority of PTF1A+ cells in the MPCs lack NKX6.1 expression, showing that PTF1A expression in progenitors is competent to suppress NKX6.1 [40]. Likewise, exogenous PTF1A also represses the expression of both SOX9 and NGN3 to completely block endocrine cell differentiation [40]. The antagonist action between Nkx6 and Ptf1a only works during a competence window before E14 in mice, where Ptf1a^+^ cells are still multipotent [40]. After this time window, the tip progenitors are irreversibly committed to an acinar fate and trunk progenitors to an endocrine or ductal fate [28, 41]. In humans, the expression pattern of NKX6.1 and PTF1A in the tip and trunk progenitors of the developing human pancreas is similar to that of mice [33], suggesting a similar mechanism. This early specification event mediated by cross-antagonism between NKX6.1 and PTF1A may represent an intrinsic mechanism by which the relative numbers of newly differentiated endocrine versus acinar cells are predetermined during pancreas development [40].

During the MPC stage, the co-expression of NKX6.1 and SOX9 is crucial for the generation of NGN3^+^ EPs that mature into β cells. In humans, the inhibition of SOX9 expression in fetal islet epithelial cells between weeks 14 and 16 has significantly decreased the mRNA levels of NKX6.1 and their proliferative capacity [42]. Previous studies reported that the level of NGN3 expression is essential for MPCs to be directed towards the endocrine fate [43]. This highly suggests that there is an important interaction between NKX6.1 and SOX9 during MPC differentiation. However, Sox9 expression is observed in embryos lacking the expression of Nkx6.1 and Ngn3 indicating that Sox9 expression is unaffected and is upstream of both [44]. It is worth to note that SOX9 expression significantly declines during EP commitment and acinar cell specification [42].

### Role of NKX6.1 in mature pancreatic islets

NKX6.1 has been found to play a crucial role for β cell homeostasis and insulin secretion. In humans, NKX6.1 has been found to be uniquely restricted to β cells and play a significant role in pancreatic β cell development. NKX6.1 is necessary for the formation and the secretory function of insulin-producing β cells. A previous study carried out on rodents showed that Nkx6.1 expression is essential for maintaining pancreatic β cell identity in vivo and its overexpression leads to increased GSIS [45]. Another study reported that Nkx6.1 is found to directly regulate genes responsible for insulin processing such as Ero1lb and Slc30a8. Moreover, key genes controlling β cell function, glucose uptake, and metabolism are found to be regulated by Nkx6.1 expression [46]. This indicates that Nkx6.1 is a master transcriptional regulator that mediates multiple crucial β cell processes.

Furthermore, NKX6.1 plays an essential role in maintaining β cell identity. It has been found that suppression of *Nkx6.1* in adult mouse β cells leads to activation of Ngn3 expression in β cells and converts them to Sst-expressing δ cells [45], indicating a change in β cell identity due to the absence of Nkx6.1. These findings come along with the results obtained from T2D models, in which β cells are converted into other islet cells [47].

A previous report provided evidence that in mice there are synergetic and compensatory mechanisms between both Nkx6 TFs, Nkx6.1 and Nkx6.2 [48]. In mice, loss of *Nkx6.1* results in defects in β cells only but loss of *Nkx6.2* shows no defects in pancreatic islets [48]. However, knockout of both TFs, *Nkx6.1* and *Nkx6.2*, leads to a reduction in the numbers of α and β cells [48], indicating that the absence of Nkx6.2 alone can be compensated by Nkx6.1, but the Nkx6.2 cannot compensate the absence of Nkx6.1. Nkx6.1 expression is crucial during the secondary transition of the pancreas development, which is distinguished with high levels of β cell replication and development [35] (Fig. 1). Mice lacking *Nkx6.1* have a decreased pancreatic β cell number without any effect on the growth of other pancreatic islet cells [35, 36].

Although in humans, NKX6.1 is restricted to β cells, it is also involved in suppressing α cell development. It has been believed that gene expression of GCG, an α cell restricted hormone, is not regulated by non-α cell TFs. However, some reports proposed that, during development, the failure to activate β cell-specific TFs directs the α cell phenotype and thus GCG gene expression [49]. Pdx1 and Pax4 have been shown to suppress Gcg gene expression by targeting Pax6 [50, 51]. Nkx6.1 drives the β cell development and maintains its identity through participation in the suppression of Gcg expression [52]. Supporting this notion, it has been reported that Nkx6.1 overexpression leads to a decrease in the *Gcg* mRNA; however, lowering of Nkx6.1 level leads to an increase in the *Gcg* mRNA levels. This inhibitory effect of Nkx6.1 on *Gcg* expression is mainly mediated by targeting Pax6. ChIP analysis reported direct interaction of Nkx6.1 with the Gcg promoter and that Nkx6.1 competes with Pax6 for the G1 element of the Gcg promoter [49].

**Fig. 4:**
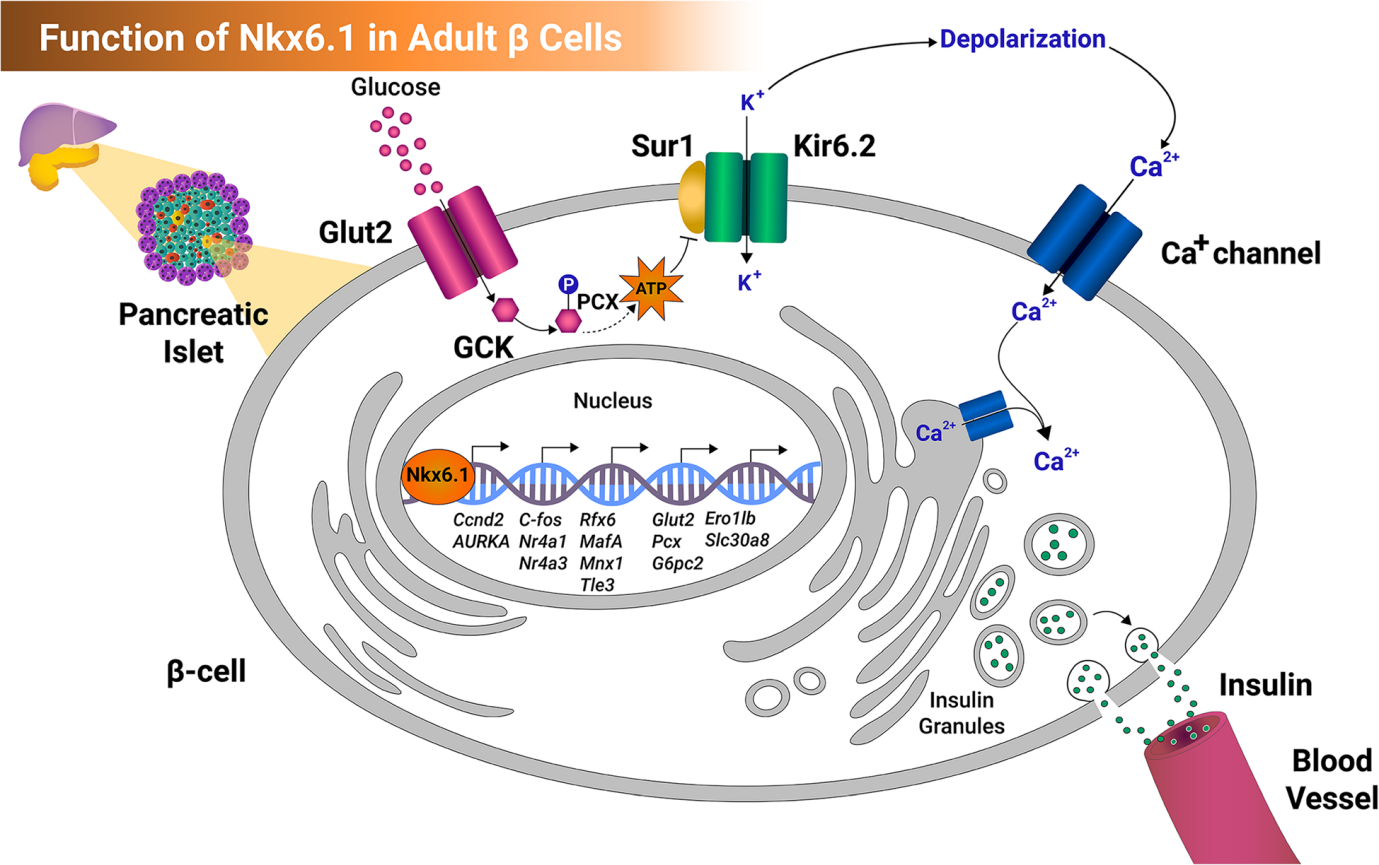
Function of NKX6.1 in adult mouse pancreatic β cells. Nkx6.1 regulates the expression of genes encoding for proteins responsible for β cell proliferation (*Ccnd2*, *Glut2*, *AURKA*, *c-Fos*, *Nr4a1*, and *Nr4a3*), β cell development and function (*MafA*, *Mnx1*, *Rfx6*, and *Tle3*), glucose uptake and metabolism (*Glut2*, *G6pc2*, *Pcx*)*,* and insulin biosynthesis (*Ero1lb* and *Slc30a8*)

There are contradictory results regarding the role of NKX6.1 in β cell proliferation. For example, forced expression of Nkx6.1 in vivo in adult mouse β cells has no effect on β cell proliferation [46, 53]; however, another study showed that overexpression of Nkx6.1 in cultured islets enhances β cell proliferation [54]. A recent study reported that *Nkx6.1* deletion in the mouse model decreases β cell proliferation through its effect on Ccnd2 [45]. These conflicting results may be due to the difference between in vivo and in vitro experiments and the change in the expression of some genes, such as Glut2, after isolating the islets from their niche [55]. Also, the absence of a positive effect of Nkx6.1 overexpression on adult mouse islets could be due to the very low rate of β cell expansion in adult mice. Interestingly, a recent study showed that Nkx6.1 activity is essential for β cell expansion postnatal [56]. Inactivation of Nkx6.1 does not block β cell proliferation before birth but it does after birth when the animal starts to eat linking Nkx6.1 role in mediating nutrient-induced β cell expansion [56]. In line with these findings, the expression of *Glut2* and *Glp1r*, nutrient sensors, are absent in β cells lacking *Nkx6.1* [56]. During the early postnatal life, a decreased β cell expansion has been observed in mice lacking *Glut2*, because glucose is important for β cell proliferation [57]. Another study demonstrated that the reduction in the intracellular glucose levels associated with *Glut2* deficiency after *Nkx6.1* inactivation leads to a decrease in the proliferative ability of mouse β cells [46], suggesting an essential role of Nkx6.1 in controlling glucose transport through *Glut2*. However, Glp1 regulates β cell proliferation through a different mechanism [58]. Therefore, the effect of *Nkx6.1* deficiency on suppressing Glut2 and Glp1r in mouse islets leads to inhibition of β cell proliferation through two different mechanisms. Forced expression of Nkx6.1 in adult rat islets leads to an increase in β cell proliferation by activating cell cycle genes, including cyclins A, B, and E and multiple kinases [54]. However, a more recent study showed that Nkx6.1 does not bind to the regulatory region of *Cyclin* genes [46]. It has been reported that Nkx6.1 directly regulates several essential pancreatic β cell genes involved in glucose uptake (*Glut2*), glucose metabolism (*G6pc2* and *Pcx*), and insulin biosynthesis (*Ero1lb* and *Slc30a8*) [46]. Furthermore, it regulates key TFs involved in the development and function of β cells [46] (Fig. 4)*.* Moreover, previous studies demonstrated that Nkx6.1 controls the expression of the nuclear receptor family 4, group A, members 1 and 3 (Nr4a1 and Nr4a3), and c-Fos, which are crucial for β cell proliferation [59, 60]. In neonatal and young mice, it has been found that loss of Nr4a1 leads to a reduction in the β cell area [59]. Taken together, these findings indicate that Nkx6.1 plays a critical role in enhancing and maintaining β cell function through regulating multiple pathways associated with insulin biosynthesis and β cell development and proliferation (Fig. 4).

There are few studies on human islets. Although Nkx6.1 knockdown impairs β cell proliferation and GSIS in rat islets [52], its overexpression in human islets results in an enhancement in β cell proliferation without improving GSIS [54]. The expression and function of some genes described above, such as GLUT2 are different in human islets; therefore, further functional studies are needed to understand the function of NKX6.1 and identify the genes regulated by NKX6.1 in human β cells.

## DNA-binding and transactivation properties of NKX6.1

Little is known when it comes to binding characteristics, transactivation properties, or the specific transcriptional targets of NKX6.1 in humans. Previous studies using animal models have provided, to a great extent, a better understanding of Nkx6.1 activity [61]. Nkx6.1 utilizes several strategies for recognizing its specific targets. Binding site selection assays have indicated that Nkx6.1 binds to a highly specific DNA sequence comprising of eight base pairs. This sequence harbors a classic 5′TAAT′3 binding core for most homeodomain factors. Furthermore, the conservancy of base pairs flanking the binding core is essential for proper identification by the Nkx6.1 homeodomain (Fig. 4). Any changes even as simple as alteration in a single base pair have a huge impact on reducing the binding affinity. The specificity of flanking sequences may contribute in narrowing down the potential targets of Nkx6.1 [61, 62]. Another strategy for NKX6.1 to identify its target is through the binding interference domain (BID). The COOH terminus domain of the homeodomain accounts for the BID of NKX6.1. BID may directly interact with the DNA-binding domain. The negatively charged COOH may interrupt the interaction between the positive charges in the DNA-binding domain and the phosphate group of the DNA (Fig. 4). The BID may provide two important features for Nkx6.1: specificity and regulation. NKX6.1 binds only weekly to DNA in vitro when a functioning BID exists. For a proper function of NKX6.1, a modification in the activity of the BID is required. Interactions between NKX6.1 and other proteins that bind nearby or are part of the transcriptional regulation complex could provide relief of interference. As an alternative, certain enzymes like kinases, phosphatases, or proteases could regulate the BID activity [62].

In general, NKX6.1 is marked as a transcriptional repressor of its targets. N-terminus of NKX6.1 has been labeled as the transcriptional repression domain, while the c-terminus (BID) has been found to be responsible for positive feedback or transcriptional activation of NKX6.1 promoter. NKX6.1 promoter harbors a sequence similar to the TAAT box and it binds to it to positively self-regulate its own expression [61, 62].

## Upstream and downstream targets of NKX6.1 associated with β cell development

**Fig. 5:**
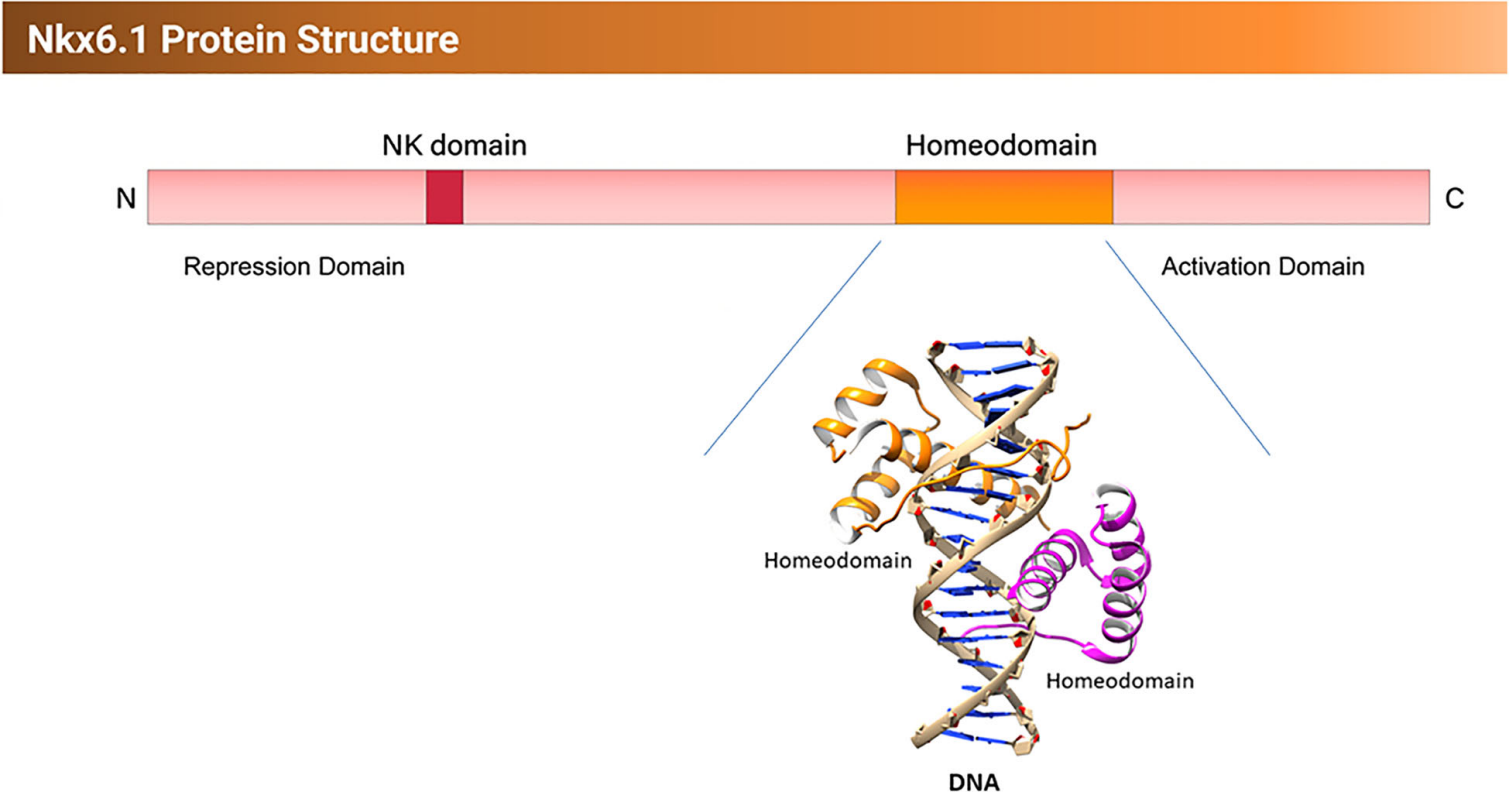
Structural architecture and domain organization of NKX6.1. A schematic representation of NKX6.1, consists of N-terminal repression domain (N), a DNA-binding homeodomain, and a C-terminal activation domain (C). Crystal structure of Aristaless and Clawless homeodomains of drosophila in complex with the DNA (PDBID: 3A01). The two homeodomains (orange and cyan) cooperate in binding onto the DNA (gray). The drosophila homeodomain structure is the closest to NKX6.1 homeodomain

Previous reports revealed that there are several NKX6.1 targets associated with multiple functions in β cells (Fig. 5). Nr4a1 and Nr4a3 have been reported as one of the main targets of NKX6.1-mediated β cell proliferation [59]. Likewise, c-Fos is a TF that is regulated by Nkx6.1 expression in rat insulinoma cells. Interestingly, Nr4a1 and Nr4a3 have been found to be downstream targets of c-Fos. This implies that the β cell proliferation induced by Nr4a1 and Nr4a3 is stimulated by Nkx6.1-mediated c-Fos upregulation [60]. Furthermore, ChIP-Seq analysis showed that Nkx6.1 directly controls the expression of important β cells and insulin processing genes, including *Glut2*, *G6pc2*, *Pcx*, *Ero1lb*, and *Slc30a8* [46]. It also controls the expression of TFs of β cell development, including *Rfx6*, *MafA*, *Mnx1*, and *Tle3* [46]*.* A previous study reported that Nkx6.1 controls cell cycle regulators, cyclins and cyclin-dependent kinases [54]; however, a recent study used ChIP-seq analysis demonstrated that the Cyclin genes are not regulated by Nkx6.1 [46]. Another target that is associated with NKX6.1-mediated β cell proliferation is Aurora Kinase A (AURKA). NKX6.1 binds to AURKA promoter, and it has been shown that AURKA is directly induced by the overexpression of Nkx6.1 in rat primary islets that resulted in the degradation of the cell cycle regulator p53 [63].

Furthermore, it has been found that NKX6.1 regulates the expression of hepatocyte nuclear factor 1 α (HNF1α) that is expressed in both hepatocytes and during pancreas development [64]. HNF1α is a key determinant of pancreas specification [13]. Although HNF1α promoter’s regulation in hepatocytes has been thoroughly studied, the mechanism by which HNF1α promoter is regulated during pancreatic development needs further investigations [65]. Transcription is regulated through a TATA-like box and proximal HNF4α-binding site. The HNF1α is fully active in hepatocytes but not in INS-1 rat insulinoma cells, providing evidence that its regulation may be controlled by alternative elements in other tissue types. Several TFs specific for β cells have been screened using transient transfection assays in 3T3 cells with co-transfection of the HNF1α-luciferase reporter [64]. A previous study reported that in β cells, NKX6.1 is an important regulator for HNF1α through its binding to the HNF1α promoter [64]. Binding site mutation analysis showed that 5′-TAAT-3′ is a real NKX6.1-binding site, which is involved in the initiation of HNF1α transcription. NKX6.1 activates HNF1α in a concentration-dependent manner [64]. Overexpression of NKX6.1 in NIT1 β cells leads to a marked increase in the expression of endogenous HNF1α by 3-fold compared to control. On the other hand, knockdown of NKX6.1 using specific siRNA significantly reduces HNF1α endogenous expression by 80% compared to the control. This provides further evidence that HNF1α is regulated by NKX6.1 [64].

Little is known about the pathways controlling the expression of NKX6.1 during β cell development, particularly in humans. It has been suggested that PDX1, a known activator of gene expressions, has NKX6.1 as one of its target genes during pancreatic β cell development [66, 67]. In *Pdx1*-null mice, the Nkx6.1 expression is lost in the dorsal pancreatic epithelium. However, the cells expressing Gcg in the dorsal pancreas bud in earlier stages still show scanty expression of Nkx6.1 [34]. This means that the Nkx6.1 production in those Gcg/Nkx6.1 double-positive cells is independent of Pdx1 [34]. hPSC studies investigating the binding sites of PDX1 during human pancreatic development have been mainly examining its role during the early MPC stage, which does not express NKX6.1 [68, 69]. However, ChIP-seq analysis on primary adult human β cell reported that NKX6.1 is among the PDX1 binding sites [68] indicating that PDX1 controls NKX6.1 in human β cells. Furthermore, NGN3 has been shown to be indirectly inducing the expression of NKX6.1 via activating PAX4. NGN3 has been found to bind to the regulatory region of PAX4 to mediate its expression in endocrine progenitors [37]. Overexpression or knockdown of SOX9 in fetal human islets has significantly affected NKX6.1 expression and subsequently INS mRNA levels [42]. This suggests that SOX9 is important for NKX6.1 expression.

Based on the hierarchy of TFs directing pancreatic development in the mice model, Nkx6.1 lies downstream of Nkx2.2 [13]. This has been proven when single mutation in *Nkx6.1* did not result in a complete block of β cell differentiation unlike in *Nkx2.2* mutant embryos [35]. This is mainly due to the fact that even though Nkx6.1 function is impaired, Nkx2.2 expression is maintained in the Nkx6.1 mutant pancreas [35]. Another study reported that Nkx6.1 is directly regulated by Nkx2.2 [67]. This eminently suggests that Nkx6.1 functions downstream of Nkx2.2 in mouse β cell differentiation [35]. Of note, in rodents, Nkx2.2 expression has been detected in the MPCs prior to the development of EPs [70], while in humans, NKX2.2 has not been found in MPCs of the fetal pancreas and starts to be expressed after endocrine induction [13]. Taken together, these findings suggest a species difference in the expression and function of NKX6.1 during pancreatic development (Table 1). NKX2.2 is expressed in both α- and β cells. In β cells, NKX2.2 preferentially binds to the Arx promoter. The methylation state of the Arx promoter might influence this binding specificity via modifications induced by DNMT3A, which is expressed in both α- and β cells indicating that the preferential recruitment of DNMT3A to the Arx promoter reckons on a β cell-specific factor such as NKX6.1. As supporting evidence, NKX6.1 has been found to bind to the Arx promoter during β cell specification [71]. Further human studies are needed to clarify the relationship between NKX6.1 and other TFs during early human pancreas development.

**Table 1:** NKX6.1 expression and function during pancreas development in rodents and humans

Developmental stage/specific role	NKX6.1 expression and function	
	Rodents	Human
Expression in the pancreatic epithelium	- At E9.5, Nkx6.1 starts to be expressed in the pancreatic epithelium [28].	- At G6.5w, NKX6.1 is first detected in dorsal buds [19].
	- At E10.5, Nkx6.1 and Ptf1a are co-expressed in the majority of MPCs.	
	- At E12.5, Nkx6.1 becomes exclusively confined in the trunk cells [28].	- At ~G7w, the NKX6.1 is co-localized with the early acinar marker, GATA4 in the MPCs giving rise to acinar cells [19].
		- At ~G9w, NKX6.1 is detected in the tip cells before losing the expression a week later and becoming confined to the trunk area [19].
Expression in endocrine cells	- At E13.5, the appearance of the first Ins^+^ cells co-expressing Nkx6.1 [30].	- In adult islets, the NKX6.1 is exclusively expressed in β cells [19].
	- Ngn3 and Nkx6.1 co-expression at the endocrine progenitor stage is crucial for their commitment to β cell lineage [38].	
		- hESC-derived β cells arise from NKX6.1^+^ endocrine progenitors co-expressing NGN3 [33, 72–74].
	- Nkx6.1 is exclusively expressed in mature β cells [30].	
Expression in ductal cells	- Ductal cells mature from NKX6.1^+^ progenitors [41].	- hESC-derived MPCs expressing NKX6.1^+^/SOX9^++^ represent ductal progenitors [24].
Role in β cell maturation and function	- Nkx6.1 directly controls the expression of β cells and insulin processing genes (*Glut2*, *G6pc2*, *Pcx*, *Ero1lb*, and *Slc30a8*) and controls the expression of TFs of β cell development, including *Rfx6*, *MafA*, *Mnx1*, and *Tle3* [46].	- In human islets, NKX6.1 overexpression does not improve on GSIS [54].
		- hPSC-derived MPCs co-expressing PDX1 and NKX6.1 differentiate into functional β cells in vitro and in vivo [18, 33, 72–74].
	- Nkx6.1 expression is required for GSIS [45, 46].	
		- hPSC-derived pancreatic progenitors lacking PDX1 (PDX1^−^/NKX6.1^+^) generate mature β cells [75].
Role in β cell identity	- Nkx6.1 suppresses Ngn3 in adult β cells to their main identity and prevents their conversion into Sst-expressing δ cells [45].	- NKX6.1 expression does not co-localize with SST, PP, or amylase during early or adult pancreas development [19].
	- Nkx6.1 suppresses Gcg expression by targeting PAX6 [52].	
		- *ARX* and *GCG* genes are completely absent in hiPSC-derived β cells that express NKX6.1 [76].
Role in β cell proliferation	- Nkx6.1 controls the expression of Nr4a1, Nr4a3, and c-Fos, which are crucial for β cell proliferation [59, 60].	- In human islets, overexpression of NKX6.1 enhances β cell proliferation [54].
	- Nkx6.1 controls the expression of AURKA, which suppresses p53 [63].	
	- Nkx6.1 controls the expression of Glut2 and Glp1r, which are crucial for β cell proliferation [46].	

*CS13* Carnegie stage 13 of embryology (~ 6.5 weeks of gestation), *CS19* Carnegie stage 19 of embryology (~ 9 weeks of gestation), *E9.5* embryonic day 9.5, *G6.5W* 6.5 weeks of gestation, *G9W* 9 weeks of gestation, *GSIS* glucose-stimulated insulin secretion, *hESCs* human embryonic stem cells, *hiPSCs* human induced pluripotent stem cells, *MPCs* multipotent progenitor cells, *TF* transcription factor

## NKX6.1 and diabetes mellitus

T2D is characterized by impaired insulin sensitivity of insulin target tissues and reduced insulin secretion by pancreatic β cells. Several lines of evidence suggest that cellular processes defect compromise β cell function and lead to T2D development [15]. Moreover, high levels of glucose in the blood have been linked with impaired expression of genes that are responsible for β cell identity [77]. Talchai et al. described that a loss of β cell features is distinguished by a drop-in insulin production, adoption of progenitor-like features, and conversion of fate into other endocrine cell types in mouse models of T2D, suggesting that loss of the β cell-differentiated state contributes in the failure of β cell in T2D [47]. However, whether loss of β cell functional properties and loss of β cell identity are linked during T2D progression remains elusive. The reduced expression in key transcriptional regulators justifies the simultaneous loss of β cell function and identity [47].

Mounting evidence suggests that NKX6.1, the β cell-enriched TF, may have a prominent role in the development of T2D. Three genome-wide association studies (GWAS) reports have identified NKX6.1 variants that are associated with T2D in East Asian individuals [76, 78, 79]. Also, decreased expression of NKX6.1 has been shown to be involved in the development of T2D in humans and rodents [47, 80]. Moreover, in vitro studies in β cell lines and isolated islets suggest a possible role for NKX6.1 in the regulation of β cell proliferation as well as of GSIS [52, 54]. A previous study reported that Nkx6.1 is necessary and sufficient to de-differentiate β cells into immature endocrine precursors in the embryo, supporting the notion that NKX6.1 maintain the differentiated state of adult β cells [45]. Together, all these evidences suggest that NKX6.1 is a key regulator of β cell function and identity and suggest its involvement in diabetes development.

It has been found that the loss of Nkx6.1 causes rapid-onset diabetes due to defects in insulin biosynthesis and secretion without affecting on β cell survival [46]. In *NkX6.1*-deficient islets, it has been found that the expression of Zinc transporter Slc30a8 (oxidoreductase Ero1lb) associated with T2D is dramatically reduced [46]. Furthermore, a severe reduction in the expression of the Pcsk1 (PC1), which converts proinsulin to insulin, is noticed in *NkX6.1*-deficient mouse islets [46]. The observed loss in insulin production and β cell functional properties is later accompanied by abnormal activation of δ cell genes in β cells. Thus, by impairing β cell function and destabilizing β cell identity, reduced NKX6.1 levels, as seen in T2D, could contribute to the pathogenesis of T2D [46].

Furthermore, it has been demonstrated that NKX6.1 is a major regulator of HNF1α and that this regulatory system is unique to β cells [64]. Mutations in the *HNF1α* gene leads to maturity-onset diabetes of the young type 3 (MODY3), the most common form of MODY, suggesting that NKX6.1 defect may be associated with diabetes development by controlling the expression of MODY3 gene. This opens the door to study the potential of novel regulatory elements that should be included when screening for mutations affecting HNF1α expression. NKX6.1, which is uniquely expressed in mature β cells of the pancreas, may implicate a new target for diagnosis of MODY [64].

## The use of human pluripotent stem cells (hPSCs) to understand the role of NKX6.1 during human β cell development

hPSCs (hESCs and hiPSCs) have become a solid approach that benefits studies on developmental biology. An outstanding amount of knowledge about pancreas development has been collected using animal models. However, despite being a very powerful tool in understanding the human pancreas, there are some limitations due to differences between mice and human species. The use of primary human β cells could serve as an alternative approach; nonetheless, being based on isolation of islets from cadaveric organ donors, its scarce availability, and poor maintenance in vitro had made it a limited option. Furthermore, it is not a suitable model to study developmental stages of the pancreas [15]. The role of NKX6.1 in β cell development has been examined with the use of hPSCs. The combination of hPSCs with single cell transcriptome analysis has revealed that β cell arises from a heterogenous population and subpopulations during differentiation [72]. However, despite the promising advances of the β cell differentiation protocols using hPSCs, there are still many shortcomings in comparison to human primary β cells [13, 15]. This indicates the urgent need to develop the existing differentiation protocols. The use of hPSCs expressing a fluorescent reporter gene can facilitate the isolation and purification of specific cell types in heterogeneous populations. Hence, the generation of genetically modified hPSC lines reporting the expression of key β cell genes including NKX6.1 will improve their differentiation towards functional β cell lineage. Furthermore, it will also help in characterization and studying the trajectory leading to this fate [81]. Likewise, the use of specific cell surface markers labeling cells of interest is beneficial to purify and isolate the hPSC-derived (PDX1^+^/NKX6.1^+^) MPCs from the produced heterogenous population (reviewed in [15]). Surface markers including GP2 and CD142 have been identified as markers distinguishing MPCs that give rise to functional β cells [33, 73, 74]. Similarly, CD49a has been identified as a marker for mature β cells co-expressing NKX6.1 and insulin [72]. Nonetheless, whether the abovementioned surface markers successfully capture all the cells expressing NKX6.1 at the defined stages remain debatable.

Interestingly enough, the use of hPSCs to study the role of NKX6.1 has unfolded the presence of another pathway by which NKX6.1^+^ MPCs progress to β cell lineage. Our recent studies reported the generation of hPSC-derived MPCs, which do not express PDX1 and express high levels of NKX6.1 (PDX1^−^/NKX6.1^+^) [82, 83]. The newly reported population (PDX1^−^/NKX6.1^+^) do not express endocrine markers, CHGA and NGN3, and ductal epithelium marker, Cytokeratin 19 [82]. These findings suggest the presence of two distinguished populations of NKX6.1^+^ MPCs during human pancreas development, in contrast to the idea of the existence of a single NKX6.1^+^ pancreatic progenitors always co-expressing PDX1 [82]. Interestingly, we have recently generated functional β cells from PDX1^−^/NKX6.1^+^/NESATIN^+^ MPCs and the generated β cells co-express PDX1 and C-peptide, indicating that there is another way to generate pancreatic β cells without going through PDX1^+^ MPCs [75]. Further hPSC-based studies are needed to understand the mechanism underlying the function of NKX6.1 during human β cell development.

## Conclusion and future perspectives

Although NKX6.1 has been reported to have a major role in the differentiation and development of pancreatic β cells, the exact underlying mechanism remains largely unknown particularly in human islets. In this review, we have summarized the crucial function of NKX6.1 that commences during MPC specification and ends with defining and maintaining the features comprising a functional β cell. NKX6.1 expression in PDX1^+^ MPCs is critical for driving them towards the right pathway. PDX1^+^/NKX6.1^+^ MPCs are merely the sole precursors that give rise to mature β cells. At the MPC stage, the antagonistic interaction between NKX6.1 and PTF1A pushes the MPCs to either endocrine or exocrine fate with NKX6.1 favoring the endocrine lineage. In regard to the NKX6.1 role in mature β cells, several studies demonstrated that NKX6.1 is essential for the insulin biosynthesis, β cell identity, and enhanced proliferation of β cells. Nkx6.1 enhances β cell proliferation through its effect on cell proliferation genes, such as *Ccnd2*, *Glut2*, *AURKA*, *c-Fos*, *Nr4a1*, and *Nr4a3*. Furthermore, Nkx6.1 plays a key role in β cell development and function by controlling the expression of key TFs, such as *HNF1α*, *MafA*, *Mnx1*, *Rfx6*, and *Tle3.* Moreover, it plays an essential role in glucose uptake and metabolism and insulin biosynthesis through its effect on *Glut2*, *G6pc2*, *Pcx*, *Ero1lb*, and *Slc30a8* (Fig. 4)*.* Taken together, this indicates that NKX6.1 utilizes many pathways for expanding and maintaining the functional β cell mass. Since most of the data described here are obtained from animal studies, we cannot apply them to humans because there are physiological differences between mice and humans. For example, one of the results obtained from mouse studies showed that Nkx6.1 regulates β cell proliferation through its effect on Glut2 [46], which is not the main glucose transporter in human β cells. This indicates that more studies on human islets and hPSC-derived β cells are needed to understand the exact role of NKX6.1.

The crucial role of NKX6.1 has pushed scientists to further dissect the exact mechanism that this TF employs to restrict the MPC differentiation into the insulin-secreting β cells. As any TF, NKX6.1 is responsible for regulating the expression of a number of genes by binding to their DNA sequence. Whether this regulation is achieved by repressing or activating the target genes, the identification of NKX6.1-targeted genes is an important aspect for a better understanding of pancreas development in general and β cells in particular. Characterizing human NKX6.1 function and downstream targets throughout pancreas organogenesis will provide great insight into the mechanisms through which NKX6.1 regulates the differentiation of MPCs into functional human pancreatic β cells. Genetic manipulation by either overexpression or knockdown of NKX6.1 expression is an important tool for the gene function analysis. This approach offers a powerful tool to detect and track the signaling cues controlling MPC’s commitment to functional β cell trajectory as oppose to other endocrine cell types. ChIP-Seq assay combined with sequencing can be used for mapping the genomic location of human NKX6.1 and subsequently identify DNA-binding sites. Studies carried out in mice models using ChIP-seq technology have already identified key genes directly and indirectly controlled by NKX6.1 expression. While these studies helped in creating a feasible understanding about NKX6.1 function, however, this cannot be necessarily translated to the human model. hPSCs have offered an alternative and a powerful solution for accurate disease modeling of humans. These hPSCs can be differentiated using efficient stepwise differentiation protocols to either MPCs, endocrine precursors, or ultimately to β cells to capture the distinctive downstream targets of NKX6.1 at each stage. ChIP-Seq analysis of NKX6.1-expressing cells during the aforementioned different stages of β cell development from hPSCs will help in identifying direct NKX6.1 pancreatic targets on a genome-wide scale. Dissecting NKX6.1-mediated governance of the pancreatic β cell differentiation will undoubtedly provide new insight into the transcriptional mechanisms regulating the differentiation of MPCs into β cells. Such knowledge will be highly beneficial in optimizing current protocols for differentiating hESC/hiPSCs into functionally mature insulin^+^ cells. The ultimate goal would be to enrich and produce large amounts of NKX6.1^+^ pancreatic progenitors that in turn leads to adequate amounts of monohormonal, glucose-responsive β cells. Moreover, identifying novel surface markers specific for MPCs that are expressing NKX6.1 is advantageous. This will be of great benefit to easily isolate and purify the population of interest for subsequent differentiation and/or transplantation. Currently, hESC-derived MPCs co-expressing PDX1 and NKX6.1 are used in clinical trials for treating type 1 diabetic patients (ClinicalTrials.gov Identifier: NCT03163511, NCT02239354), indicating the significance of transplanting a pure population of MPCs expressing high levels of NKX6.1 for treating diabetic patients (reviewed in [15]).

Diabetes pathophysiology involves several factors, including β cell loss and alterations in β cell function, identity, and proliferation as a result of defects in the expression of β cell-specific TFs, such as NKX6.1. Thus, discovering treatments sustaining appropriate levels of those TFs in β cells could be one of the key strategies to treat specific forms of diabetes.

## Data Availability

Not applicable

## Abbreviations

DM: Diabetes mellitus
GCG: Glucagon
GSIS: Glucose-stimulated insulin secretion
GWAS: Genome-wide association studies
hESCs: Human embryonic stem cells
hiPSCs: Human induced PSCs
hPSCs: Human pluripotent stem cells
INS: Insulin
MPCs: Multipotent pancreatic progenitor cells
SST: Somatostatin
TFs: Transcription factors
